# New UPLC–MS/MS assay for the determination of tamoxifen and its metabolites in human plasma, application to patients

**DOI:** 10.2144/fsoa-2018-0113

**Published:** 2019-03-22

**Authors:** Christine Bobin-Dubigeon, Mario Campone, Elsa Rossignol, Estelle Salaun, Marie-Bernadette Amiand, Jean-Marie Bard

**Affiliations:** 1 Biopathology Department ICO René Gauducheau, Bd J Monod, 44805 Nantes Saint Herblain Cedex, France; 2 Faculté de Pharmacie, Université de Nantes, EA 2160 MMS, IUML FR3473 CNRS, 1 rue Gaston Veil, 44000 Nantes, France

**Keywords:** analytical method, endoxifen, 4-hydroxytamoxifen, N desmethyl tamoxifen, plasma, tamoxifen, therapeutic drug monitoring, UPLC–MS/MS, validation method

## Abstract

**Aim:**

A rapid UPLC–MS/MS method for the determination of tamoxifen (TAM), *N*-desmethyltamoxifen, 4-hydroxytamoxifen and endoxifen in human plasma was validated, after a simple protein precipitation.

**Materials and methods:**

The analysis was achieved on a C^18^ analytical column, using a gradient elution with a mobile phase of water and acetonitrile for 4.5 min.

**Results:**

The validated method demonstrated good linearity between 1 and 500 ng/ml for TAM and *N*-desmethyltamoxifen; between 0.2 and 100 ng/ml for endoxifen and between 0.1 and 50 ng/ml for 4-hydroxytamoxifen. The method also provided satisfactory results in terms of within day and between day imprecisions and accuracy, and also in terms of time stability and specificity.

**Conclusion:**

The method is applied routinely for TAM monitoring from patients undergoing therapy.

## Lay abstract

Tamoxifen is a drug widely used in the treatment of hormone-dependent breast cancer. However, interindividual variations of drug concentration are described, according to the metabolization of each woman. We have developed and validated a rapid method to quantify tamoxifen and its metabolites in the plasma, using a chromatographic analytical technique coupled with mass spectrometry detection (UPLC–MS/MS). This method could routinely be used to adapt the treatment and give the right dose to the right patient.

For the last four decades in early breast cancer overexpressing estrogen receptors, tamoxifen (TAM) has been the standard therapy for nonmenopausal women, as it is able to reduce the risk of recurrence and decrease breast cancer mortality [1]. The TAM is a selective estrogen receptor modulator, which is able to competitively antagonize estrogen receptors on breast tissue. However, this drug is ambivalent concerning its target tissues with agonist estrogen effects on bone, endometrium and also liver. This explains its beneficial effects, such as increased mineral bone density [2], and also its side effects such as thromboembolic events [3], increased endometrial cancer events [4] and hot flushes [5].

The TAM (Figure 1) is mainly metabolized in the liver by two pathways. The first one is 4 hydroxylation, catalyzed by CYP2D6, resulting in the formation of the most antiestrogenic component, the 4-hydroxytamoxifen (4OHTAM) (Figure 1), as has been shown *in vitro* since 1977 [6,7]. However, its contribution to TAM effect is considered low, at less than 10%.

**Figure 1.**
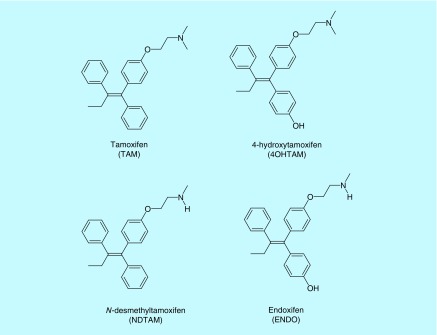
**Structures of tamoxifen and its main derivatives.**

Approximately 92% of TAM metabolism is due to *N*-demethylation to form *N*-desmethyltamoxifen (NDTAM; Figure 1), which is then metabolized to a number of molecules, such as endoxifen (ENDO; Figure 1), formed after hydroxylation by CYP2D6, but also demethylation by CYP3A4 from 4OHTAM [8]. Overall, more than 22 metabolites of TAM have been identified.

The TAM could be considered as a prodrug, as its metabolites are more active than the drug itself. The antiestrogenic activities of ENDO and 4OHTAM are similar [9], but plasmatic concentrations of ENDO in patients receiving TAM therapy are fivefold higher than that of 4OHTAM [10], with large interpatient variability [11], mainly due to the polymorphisms of the main cytochrome p450 enzyme CYP2D6. More than 100 genetic variants have been described [12], resulting in a wide range of enzyme activities, from low to ultra-metabolizer phenotypes. However, the impacts of CYP2D6 variants on the TAM pharmacogenomics are controversial [13]. Therefore, in a recent work [14], the clinicians were encouraged not to use CYP2D6 polymorphisms to guide adjuvant endocrine therapy selection. Recent data have suggested that low ENDO concentration and decreased CYP2D6 activities predict shorter distant relapse-free survival [15] and a threshold ENDO concentration to achieve therapeutic benefit has been suggested [10].

At the same time, the impact of TAM transporter polymorphisms needs to be explored [15].

Therefore, the availability of an analytical method to quantify plasmatic concentrations of TAM and its metabolites could be useful for therapeutic drug monitoring to improve TAM therapy.

Methods for the quantification of TAM and its main metabolites were developed in the 80s in serum [16], but also in bile fluid [17]. However, only TAM, NDTAM and 4OHTAM were quantified, as ENDO was not yet identified [16,18]. Since 2000, the importance of ENDO as a biologically active metabolite has resulted in the development of full metabolite analytical methods [19]. Quantification can be performed in rodents [20] and in human matrices such as scalp [21], tumors [18], dried blood [22] and serum [16,23,24], but more usually plasma [25–30]. For most of these methods, the separation of analytes is performed by GC [31], HPLC [16,18,19] or more recently by UPLC [21,28,32]. Various detection methods have been described, such as UV or fluorimetry [24,33], and single mass [21] or MS/MS [28,30], with different levels of sensitivity. For plasmatic quantification, various preanalytical preparations have been described: protein precipitation [16,29], solid-phase extraction [18,21] and liquid–liquid extraction [28].

According to the available clinical data, plasmatic concentrations range from 20 to 307 ng/ml [18,30], from 25 to 530 ng/ml [18], from 0.22 to 200 ng/ml [18,34] and from 0.32 to 19 ng/ml [19,34] for TAM, NDTAM, 4OHTAM and ENDO, respectively. Very few available methods could quantify quickly TAM and its three main metabolites with a satisfactory LOQ. Depending on the analytical procedures, the run times to quantify TAM and its metabolites by UPLC–MS/MS range from 6 [28] to more than 10 min [29,30]. It appears, therefore, necessary to optimize these methods and develop a rapid and sensitive method to quantify TAM and its metabolites in human plasma.

In view of future pharmacokinetic studies, we developed a method to quantify TAM, NDTAM, 4OHTAM and ENDO in plasma with a low LOD of around 0.2 to 0.5 ng/ml, with a range from 1 to 500 ng/ml for both TAM and NDTAM, and from 0.1 to 50 and 0.2 to 100 ng/ml, for 4OHTAM and ENDO, respectively. This method requires only 100 μl of plasma and involves a protein precipitation step with formic acid for the purification of plasma samples. A validation procedure, according to international guidance, was fully successfully performed.

## Experimental

### Chemicals & reagents

The TAM, NDTAM, 4OHTAM, ENDO, NDTAM-D5, OHTAM-D5 and ENDO-D5 were purchased from Toronto Research (Canada). The TAM-(*N,N*-dimethyl-13C2)-15 N was obtained from Sigma Chemical Company (Quentin Fallavier, France). Optima-grade methanol and formic acid were purchased from VWR (Fontenay sous Bois, France), acetonitrile and ammonium formate were obtained from Biosolve Chimie (Dieuze, France) and Sigma Chemical Company, respectively. Ultrapure water was provided from our Millipore system, MilliQ Plus (Molsheim, France).

### Preparation of calibrators & quality control samples

Stock solutions of studied analytes and internal standards were prepared, at 1 mg/ml of Z-isomer, in methanol. These stock solutions were diluted from 10 to 5000 ng/ml for TAM and NDTAM, from 2 to 1000 ng/ml for ENDO and from 1 to 500 for 4OHTAM in water/methanol 30/70, with formic acid 0.1%, in order to solubilize the analytes. These diluted solutions were extemporaneously further diluted in blank plasma to yield the following calibrator concentrations from 1, 5, 20, 100, 250 and 500 ng/ml; from 0.2, 1, 4, 20, 50 and 100 ng/ml and from 0.1, 0.5, 2, 10, 25 and 50 ng/ml for both TAM and NDTAM, ENDO and 4OHTAM, respectively.

Internal standard solutions were extemporaneously diluted in acetonitrile: formic acid 0.1%, for final concentration 5 and 20 ng/ml for ENDO and OHTAM and for TAM and NDTAM, respectively.

For the preparation of quality control (QC) samples, independent stock solutions were prepared as above, to yield the following concentrations in plasma: 1, 2.5, 40 and 400 ng/ml; 0.2, 0.5, 8 and 80 ng/ml and 0.1, 0.25, 4 and 40 ng/ml, for both TAM and NDTAM, ENDO and 4OHTAM, respectively.

All the stock solutions and intermediary solutions were aliquoted and stored at -80°C.

### Instrumentation

The UPLC–MS/MS analysis was conducted using an Acquity UPLC H-Class System coupled to a Xevo TQD Tandem Mass spectrometer (Waters, MA, USA). The ESI was operated in positive ionization mode. Multiple ion monitoring chromatograms were acquired using MassLynx Mass Spectrometry Software 4.1 (Waters).

### Chromatographic & mass spectrometer conditions

An Acquity UPLC BEH C18 (50 × 2.1 mm, 1.7 μm) analytical column was used. Mobile phase A was water: formic acid (100:0.5, v:v) ammonium formiate 2 mM, and mobile phase B was acetonitrile: formic acid (100:0.5, v:v). A linear gradient was ramped up from 40 to 95% of solvent B in 2.5 min at flow rate 0.6 ml/min. These conditions were maintained during 0.75 min to clean the column and initial conditions were then restored (95–40% B, 0.05 s, 40% B, 0.1 s). The temperatures were 10 and 50°C, for the autosampler and the column, respectively, and the injected volume was 7 μl. The total run time of this analysis is 4.5 min.

MS/MS was performed in the positive ion ESI mode. The dessolvation temperature, the cone gas and the ion spray voltage were 600°C, 1 l/h and 1 kV, respectively. The cone voltage was kept at +50 V for OHTAM and NDTAM-d5, and +45 V for the other studied molecules. The dwell times were 0.005 s. Nitrogen was used as the nebulizing and curtain gas (800 l/h). Collision was achieved with argon, the autosampler injector was at 10°C and the source temperature was 150°C. Multiple reactions monitoring mode was applied for the quantification, MS/MS settings are presented in Table 1.

**Table 1.** **Retention times and multiple reactions monitoring transitions for tamoxifen, it three metabolites and internal standards by UPLC–MS/MS.**

**Analyte**	**Retention time (min)**	**Parent (*m/z*)**	**Quantification transition (*m/z*)**	**Confirmation transition (*m/z*)**	**Cone voltage (V)**	**Collision energy (eV)**
Z-Endoxifen	0.95	374	58	129	45	22/25

Endoxifen-D5	0.89	379	–	152	45	21

Hydroxytamoxifen	1.01	388	72	129	50	27/29

Hydroxytamoxifen-D5	0.97	393	–	134	45	25

*N*-desmethyltamoxifen	1.93	358	58	91	45	22/36

*N*-desmethyltamoxifen-D5	1.93	363	–	134	50	28

Tamoxifen	2.01	372	72	129	45	25/26

Tamoxifen-(*N,N*-dimethyl-^13^C_2_)-15 *N.*	2.01	375	47	75	40	29/45

Calibration curves were constructed by plotting the peak area ratios of the analytes to internal standards versus the known concentrations with a weight factor of 1/concentration.

### Sample preparation

A total of 100 μl of water:formic acid 100:1 (v:v) was added to 100 μl of plasma samples in 1.5 ml microcentrifuge tubes, and vigorously vortexed during 30 s in order to remove protein interaction with plasma. Methanol (100 μl) was added and the aliquots were transversely agitated during 10 min at room temperature. The samples (300 μl) were again vortexed after the addition of 400 μl of internal standard solution and then centrifuged at 18,000 × g for 10 min at 4°C. Finally, 300 μl of supernatant was mixed with 300 μl of water: formic acid (100:0.2, v:v) ammonium formate 2 mM directly in the vials.

### Validation

The UPLC–MS/MS method was validated in agreement with the Guidance for Industry, Bioanalytical Method Validation, as specified by the US FDA [35,36] and according to EMEA guidance [37]. A full validation procedure was performed, including specificity, selectivity, linearity, within-run and between-run precision and accuracy, recovery of analytes, stability after sample preparation [38], LOD and LLOQ, the effects of dilution.

Calibration standards of seven levels (including blank) and sets of QC samples (four concentrations) were prepared.

#### Selectivity, specificity & sensitivity

Six different blank human EDTA plasma samples were processed, with and without TAM and metabolites, in order to ensure the absence of interfering peaks. The effects of the matrix on ion suppression, ion enhancement and extraction efficiency were also determined.

In a second time, at the LLOQ (0.1, 0.2 and 1 ng/ml for 4OHTAM, ENDO and both TAM and NDTAM, respectively), samples were processed in order to identify the absence of interfering peaks with and without potential interfering molecules. Zoledronic acid, acetylsalicylic acid, ranitidine, esmolol, propranolol, amitriptyline, furosemide, lidocaine, midazolam, clorazepate dipotassium, pantoprazole, acetaminophen, salbutamol and metopimazine at the final concentration of 1 μg/ml were added to plasma samples, before protein precipitation procedure, and the analysis was done using the described procedures. All these drugs were chosen in large panel pharmacologic classes as potential co-medications.

#### Recovery from plasma & mass spectrometric matrix effects

The extraction recovery for TAM, metabolites and the internal standards added to human plasma were determined at the four levels of QC, in five replicates. The extraction recovery, expressed as a percentage, was evaluated by the ratio of the area of extracted QC and the area of drug-free plasma, spiked with QC solutions, at the corresponding concentrations.

The ionization recovery, expressed as a percentage, was evaluated by the ratio of the area of drug-free plasma spiked with QC solutions, and the area of QC solutions at the corresponding concentrations, directly injected into the column. Carryover between samples was also determined.

Matrix effects were evaluated by the ratio between the area of hemolyzed plasmas or opalescent plasmas, and the area of normal plasmas, expressed in percentage for two levels of QC (QC low and QC high), in three replicates.

#### Calibration curve & sample quantification

Calibration curves were prepared in drug-free plasma spiked with working solutions of TAM and metabolites standards. The range of standard concentrations tested were from 0.1 to 50 ng/ml, from 0.2 to 100 ng/ml, from 1 to 500 ng/ml, for 4OHTAM, ENDO and both TAM and NDTAM, respectively. Six calibration curves were analyzed by weighted linear regression (1/×).

The peak area ratios of TAM and metabolites to that of respective internal standards were analyzed by linear regression to estimate the slope, intercept and correlation coefficient of the calibration curve. Standard curves in each analytical run were used to calculate the concentrations of the QC samples.

#### Intraday & interday precision & accuracy

The precision and accuracy of the assay was determined from the QC plasma samples.

To determine intraday precision and accuracy, four different concentrations of QC samples were analyzed in five replicates. Precision was expressed as the RSD% of peak area ratios for the five replicates, of each QC sample. Accuracy was evaluated by calculating the concentration of each QC sample, using the calibration curve that was obtained on the same day, and determining the relative error percentage of the measurement. The results were also expressed as the 95% CI for the mean of individual bias (95% CI of accuracy [%]).

To determine interday precision and accuracy, the QC samples were analyzed on six separate days. Precision was expressed as the RSD% for each level concentration of each QC sample, on six separate days. Accuracy was evaluated by calculating the percentage difference between expected concentration and the mean concentration of each QC sample on six separate days. Limits of acceptable intra and interday precision and accuracy were set at RSD% <15% and ±15% deviation from expected, respectively.

#### Limit of quantification & detection

The LOD was set as the lowest concentration of the analytes (TAM, metabolites and internal standards) that could be detected with a S/N ratio of 3:1. The LLOQ and LOQ were defined as the lowest concentration of the calibration samples that could be quantified, with an acceptable level of precision (RSD < 20%) and accuracy (RE% ± 20%). The LOQ samples were analyzed in quadruplicate and evaluated as unknown samples on six different days.

#### Dilution effects

The integrity of the dilution had been monitored by diluting QC samples (n = 5 replicates for each dilution) above upper limit of quantification (500, 50 and 100 ng/ml, for TAM and NDTAM, for OHTAM and for ENDO).

#### Stability of the extracted samples

The QC samples were tested for extracted sample stability (extracted samples waiting in the autosampler during 12 to 24 h), short-term room temperature conditions (4 to 12 h at room temperature with light conditions) and long-term storage conditions (-80°C). The calculated response at t = 0 h was compared with the calculated response at the different times.

#### Patients & sampling

The clinical protocol was approved by the ethics committee of Grand Ouest (Comité de Protection des Personnes Grand Ouest IV- Eudract number: 2008-007652-10). Ten women with early breast cancer (mean age and SD 63.4 ± 13.2) had been diagnosed for estrogen receptor-positive tumors and received TAM therapy 20 mg/day for at least 5 weeks. Blood samples were taken in the morning before ingesting a new dose of TAM, and between 18 and 24 h after the last TAM intake. After centrifugation (1000 × *g*, 10 min at 4°C), EDTA plasma was immediately stored at -80°C until analysis.

## Results & discussion

**Figure 2.**
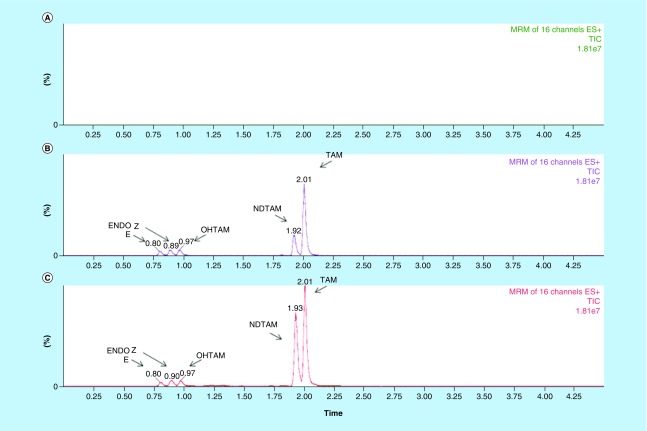
**Main chromatograms of plasmatic tamoxifen method.** Main representatives total ion current chromatograms blank extracted matrix **(A)**, extracted plasma standard containing 0.25 ng/ml OHTAM, 0.5 ng/ml ENDO, 2.5 ng/ml of NDTAM and TAM **(B)**, and an extracted plasma from patient at steady state taken daily 20 mg of tamoxifen **(C)**. ENDO: Endoxifen; NDTAM: *N*-desmethyltamoxifen; OHTAM: Hydroxytamoxifen; TAM: Tamoxifen.

Among the various tools to individualize treatment based on blood concentration, UPLC–MS/MS is widely used to optimize therapeutic effectiveness for drugs in many therapeutic classes [39]. In our work, an efficient separation of TAM and its metabolites was obtained using a classical reversed phase octadecylsilyl column C18 [24,40], according to their hydrophobicity (Figure 2: representative chromatogram). This ethylene-bridged hybrid column reduces unwanted silanol interaction increasing peak tailing. A gradient mobile phase was applied to perform adequate separation of early hydrophilic interfering matrix components as described [28]. Mass spectrometric conditions and the product ions used for identification and quantification have been widely previously described and adapted to our method [21,23,26,29,41]. ESI is the most common ionization technique, especially for polar analytes, like TAM and its metabolites. Internal standards as labeled derivatives of each specific analyte were chosen despite the use of a unique internal standard in some methods [22]. Moreover, the relative efficiency of ionization of analytes and its internal standard should not be affected by using stable isotope-labeled analogs.

The sample pretreatment is a crucial step of an analytical method. Of course, SPE [18] and liquid–liquid extraction [19,21,28] improve the purification of extracts. However, manual SPE could be of poor reproducibility and expensive, and liquid–liquid extraction requires adapted evaporation equipment. Therefore, protein precipitation by a mixture of acetonitrile: formic acid 0.1% was optimized to allow satisfactory results in terms of extraction and ionization recoveries, but also practical aspects and low costs.

Including all these adaptations, this new rapid and easily method was successfully validated.

### Specificity & selectivity

Under optimized UPLC–MS/MS conditions, TAM and metabolites were separated with retention times of 0.93, 1.01, 1.96 and 2.04 min for ENDO, 4OHTAM, NDTAM and TAM, respectively (Figure 2). In order to demonstrate the specificity of the method, ten blank human plasmas were injected. No significant interfering peak was detected at the retention times of analytes of interest.

Studied co-medications at the final concentration of 1 μg/ml did not modify TAM, TAM metabolites and internal standards responses. Indeed, the peak areas of studied analytes were not modified, even at the lowest concentrations, by the presence of co-medication substances. The TAM is a long-term treatment (over 5–10 years), and patients are able to use a broad range of pharmacological molecules during this period. Therefore, we chose to test the impact of various molecules such as psychotropes and antihypertensive drugs. So, according to Guidance for Industry Bioanalytical Method Validation [35], our method is specific.

### Limit of quantification & detection

With our method, the lowest LOQ is 0.1 ng/ml for 4OHTAM, 0.2 ng/ml for ENDO and 0.5 ng/ml for both TAM and NDTAM. The LOD is 0.1, 0.2, 0.5 and 0.5 ng/ml for 4OHTAM, ENDO, AM and NDTAM. Most previous works described quantification with higher LOQ especially for 4OHTAM and ENDO (around 0.2 and 0.5 ng/ml for [26,28], respectively).

### Linearity

The calibration curves were obtained by plotting the ratio of the peak area of studied analytes to each specific internal standard against the respective concentration. All calibration curves proved to be linear over the concentration range of 1–500, 0.1–50 and 0.2–100 ng/ml for both TAM and NDTAM, 4OHTAM and ENDO, respectively when evaluated by weighted (1/×) linear regression. Tables 2 and 3 show the mean deviation (RE) and the RSD at each calibration level, calculated using data obtained on six consecutive runs, for the four analytes. The criterion for accepting any curve was that all data points should have an RSD and an RE of less than 15%. The results of the lowest standard point were -3.2 and 8.4%; -7.3 and 1.6%; -3.0 and 6.7%; -7.7 and 6.4% for RE and RSD, for TAM, NDTAM, 4OHTAM and ENDO, respectively.

**Table 2.** **Assayed concentrations of calibration standards of tamoxifen and N-desmethyltamoxifen.**

	**Nominal concentration (ng/ml)**	**Assayed concentration**						**Mean**	**RE (%)**	**RSD (%)**

		**Run 1**	**Run 2**	**Run 3**	**Run 4**	**Run 5**	**Run 6**			
	1	1.01	0.87	0.88	0.97	0.99	1.08	0.97	-3.2%	8.4%

	5	5.0	5.4	4.7	4.9	4.9	4.8	4.9	-0.7%	4.7%

TAM	20	19.9	21.5	22.1	20.1	19.7	19.8	20.5	2.6%	4.9%

	100	100.0	100.0	107.2	103.8	105.6	90.4	101.1	1.2%	5.9%

	250	247.7	239.5	255.4	256.6	246.3	268.7	252.4	0.9%	4.0%

	500	502.4	508.7	485.7	489.7	498.5	491.1	496.0	-0.8%	1.8%

	Slope	0.0073672	0.0049791	0.0068426	0.0067050	0.004933	0.0048634			

	*Intercept*	7.060e-005	0.00092572	0.000926442	0.00068075	-2.90343e-005	-0.0005760			

	r^2^	0.999960	0.999112	0.998480	0.0752934	0.999558	0.997080			

	1	0.95	0.91	0.92	0.92	0.94	0.93	0.93	-7.3%	1.6%

	5	5.12	5.12	4.94	4.99	5.13	5.36	5.11	2.2%	2.9%

NDTAM	20	19.86	20.92	20.70	20.05	19.70	20.63	20.31	1.6%	2.5%

	100	101.25	105.69	104.84	111.77	105.33	93.62	103.75	3.7%	5.8%

	250	262.15	238.08	262.19	243.65	253.89	259.42	253.23	1.3%	4.0%

	500	486.67	505.29	482.41	494.62	491.01	496.03	492.67	-1.5%	1.6%

	Slope	0.0356403	0.0197391	0.0313202	0.0297525	0.0206491	0.0213804			

	*Intercept*	-0.00370052	0.00547332	0.00188986	0.00272543	8.88547e-005	-0.0035837			

	r^2^	0.998859	0.998821	0.998257	0.998103	0.999389	0.999005			

NDTAM: *N*-desmethyltamoxifen; RE: Relative error; TAM: Tamoxifen.

**Table 3.** **Assayed concentrations of calibration standards of hydroxytamoxifen and endoxifen.**

	**Nominal concentration (ng/ml)**	**Assayed concentration**						**Mean**	**RE (%)**	**RSD (%)**

		**Run 1**	**Run 2**	**Run 3**	**Run 4**	**Run 5**	**Run 6**			
OHTAM	0.10	0.10	0.09	0.11	0.09	0.09	0.10	0.10	-3.0%	6.7%

	0.50	0.53	0.53	0.46	0.50	0.51	0.49	0.50	0.5%	4.7%

	2	1.90	2.11	1.93	2.08	2.07	2.05	2.02	1.2%	4.3%

	10	10.36	9.85	10.65	10.63	10.22	9.56	10.21	2.1%	4.2%

	25	25.54	24.73	24.22	24.44	25.65	24.76	24.89	-0.4%	2.3%

	50	49.18	50.29	50.24	49.87	49.06	50.63	49.88	-0.2%	1.3%

	Slope	0.100984	0.0556617	0.0897014	0.0752934	0.0573051	0.0582117			

	*Intercept*	-0.0023617	-0.0004412	-0.00177371	0.00028937	-0.00163476	-0.00146074			

	r^2^	0.999483	0.999817	0.999133	0.999343	0.999500	0.999637			

ENDO	0.2	0.18	0.18	0.20	0.20	0.18	0.17	0.18	-7.7%	6.4%

	1	1.05	1.06	0.86	1.02	1.03	1.12	1.02	2.3%	8.3%

	4	4.11	4.15	4.41	3.83	3.99	4.41	4.15	3.7%	5.5%

	20	20.95	20.63	20.73	20.49	20.81	17.87	20.25	1.2%	5.8%

	50	49.99	47.95	50.30	51.21	53.97	51.73	50.86	1.7%	3.9%

	100	98.91	101.23	98.69	98.46	95.23	99.91	98.74	-1.3%	2.0%

	Slope	0.0876157	0.0480468	0.0806377	0.0658652	0.0496336	0.0501334			

	*Intercept*	0.00179152	0.0010967	-0.00331261	-0.0004144	8.885e-005	-0.00368334			

	r^2^	0.999613	0.999234	0.999378	0.999570	0.996606	0.997968			

ENDO: Endoxifen; OHTAM: Hydroxytamoxifen; RE: Relative error.

The regression coefficients (r^2^) for each calibration curve were >0.997.

Calibration curves described in the literature for TAM and NDTAM range between 5 and 1000 ng/ml [23,41]. The dilution process of upper limit of quantification (1/2, 1/5, 1/10 and 1/100) was performed and led to acceptable results (RSD and an RE of less than 15%). For the most biological active metabolite, ENDO, the first points of calibration curve were usually up to 0.5 ng/ml [25]. Therefore, the calibration curve obtained with this method is suitable for low TAM metabolites plasma concentration determination.

### Precision & accuracy

Within-run and between-run precision and accuracy were determined with QC samples at different concentrations as described in the experimental section. Data for within batch and between batch precision and accuracy of the method are presented in Table 4.

**Table 4.** **Within-run and between-run precision and accuracy of the quantification of tamoxifen, *N*-desmethyltamoxifen, hydroxytamoxifen and endoxifen.**

**Analytes**	**Nominal concentration (ng/ml)**	**Within-run precision (%)**	**Between-run precision (%)**	**Accuracy (%)**	**95% CI of accuracy (%)**
Tamoxifen	1	11.0	9.0	-2.7	-5.9–0.42

	2.5	5.8	6.1	2.7	-1.1–3.6

	40	4.1	5.1	4.7	1.4–5.3

	400	5.3	5.3	3.7	0.6–4.2

*N*-desmethyltamoxifen	1	12.4	9.3	-5.2	-6.7- (-0.9)

	2.5	6.4	7.9	-2.3	-6.0- (-0.5)

	40	4.5	5.6	3.0	0.2–4.4

	400	11.5	5.9	4.1	1.4–5.7

Hydroxytamoxifen	0.1	12.2	10.3	-0.2	-4.2–3.4

	0.25	11.1	8.8	-1.6	-4.3–2.0

	4	4.2	4.2	8.7	6.5–9.8

	40	8.2	4.7	10.1	7.4–10.7

Endoxifen	0.2	11.0	8.9	0.8	-3.5–3.2

	0.5	11.1	8.2	-2.0	-4.7–0.2

	8	7.1	6.4	2.9	0.8–5.5

	80	6.1	5.1	2.6	0.6–4.3

Within-run precision ranged between 4.1 and 11.0%, 4.5 and 12.4%, 4.2 and 12.2%, 6.1 and 11.1%; between-run precision ranged between 5.3 and 9.0%; 5.6 and 9.3%; 4.7 and 10.3%, and 5.1 and 8.9 % and the range of accuracy was -2.7 to 4.7, -5.2 to 4.1%, -0.2 to 10.1% and -2.0 to 2.9%, for TAM, NDTAM, 4OHTAM and ENDO, respectively. No carryover effects were observed.

### Recovery from samples

The mean relative ionization recovery for the studied analytes and their respective internal standards ranged between 94.0 and 103.1% for NDTAM and ENDO-D5. The mean percent of extraction recovery of TAM, metabolites and internal standards from plasma was also evaluated and ranged between 95.7 and 109.4% leading to a global recovery range between 99.8 and 124%. These results indicate good recovery and low ion suppression. As expected, the impact of hemolyzed blood on analytical performance was evaluated. The presence of hemolyzed blood in plasma has an impact on the quantification, as described for other molecules such as atorvastatin and carvedilol [42]. These results indicate good recovery and allowed us to conclude that our method is able to quantify TAM and associated metabolites in human plasma samples, except for hemolyzed blood plasma.

### Stability

The results of stability testing on extracted samples are summarized in Table 5. Samples were considered stable if the loss of concentration was lower than 15%. Three QC samples were prepared and processed in quadruplicate at t = 0 h with the calibration row. The concentrations of TAM and metabolites were determined in plasma over the 4 h period tested. The extracted solutions were stable for at least 24 h when kept in the instrument rack inside the auto sampler, maintained at 15°C, as the estimated loss of concentration is lower than 15%. As previously described by [26,28], no degradation was observed when exposed to daylight.

**Table 5.** **Stability of tamoxifen, N desmethyltamoxifen, hydroxytamoxifen and endoxifen in plasma (short-term stability), after extraction (extracted short-term stability) and after freeze–thaw cycle.**

**Analytes**	**Nominal concentration (ng/ml)**	**Short-term stability (%RSD)**	**Extracted short-term stability (%RSD)**	**Freeze-thaw cycle stability (%RSD)**
Tamoxifen	2.5	-4.1	6.5	-6.6

	400	2.5	8.1	-7.9

*N*-desmethyltamoxifen	2.5	-3.4	0.7	-7.7

	400	6.1	4.5	-8.4

Hydroxytamoxifen	0.25	6.8	0.3	9.0

	40	12.7	5.7	7.0

Endoxifen	0.5	2.4	-3.9	12.8

	80	7.9	9.7	-0.8

### Assay application

This UPLC–MS/MS method was applied to the quantitation of TAM and its main metabolites in TAM-treated breast cancer patients. As shown in Table 6, the concentrations ranged from 108 to 330 ng/ml for TAM, 152 to 329 ng/ml for NDTAM, 0.91 to 2.63 for OHTAM and 3.55 to 15.21 for ENDO. Like previous published data [28], the interindividual variations of plasma TAM and metabolites concentrations were important (between 22.2 and 40.9%). The impact of CY2D6 polymorphism could in part explain these interindividual variabilities.

**Table 6.** **Steady-state concentrations of tamoxifen and its three metabolites in plasma collected from breast cancer patients receiving 20 mg tamoxifen once daily (n = 10).**

**Patients**	**Tamoxifen (ng/ml)**	***N*-desmethyltamoxifen (ng/ml)**	**Hydroxytamoxifen (ng/ml)**	**Endoxifen (ng/ml)**
1	250	261	2.14	11.35

2	144	242	1.14	12.58

3	108	152	0.91	4.01

4	330	280	2.63	11.04

5	217	326	2.54	15.21

6	236	328	1.59	9.69

7	176	290	2.05	9.84

8	134	190	1.15	5.62

9	195	285	2.02	3.55

10	262	329	1.14	12.22

Mean	205	268	1.73	9.51

RSD (%)	32.99	22.19	36.24	40.88

## Conclusion

An UPLC–MS/MS method to quantify TAM and its metabolites in plasma was developed and validated. The UPLC–MS/MS was found to be more sensitive, rapid and selective than previously reported methods. Good accuracy and precision were also achieved. The extracted plasma samples were found to be stable for up to 24 h. Many common drugs did not influence the determination of these analytes in human plasma. Our analytical method has the advantage of a reduced runtime, as well consuming little time and money, without SPE extraction. This method could, therefore, be easily used for pharmacokinetics studies in clinical trials, for TAM drug monitoring and adapted TAM schedule administration, especially for poor or enhancer CY2D6 metabolizers.

## Future perspective

In the perspective of personalized medicine, therapeutic drug monitoring of TAM treatment by using a rapid method should be the standard in the future. Our method could be a tool to enable routine performance of these treatment optimizations.

**Summary points**

- A new analytical method to quantify by UPLC–MS/MS of tamoxifen (TAM) and its metabolites in plasma was developed.
- This method included a simple preparation step by protein precipitation, using acetonitrile and methanol.
- The TAM, endoxifen, *N*-desmethyltamoxifen and 4-hydroxytamoxifen were separated on a UPLC C^18^ column and monitored by MS–MS detection.
- This validated method was rapid and sensitive.
- It was applied to routinely monitor the steady state plasma exposure of TAM and its metabolites in hormone-dependent breast cancer.

## References

[1] Gierach GL, Curtis RE, Pfeiffer RM (2017). Association of adjuvant tamoxifen and aromatase inhibitor therapy with contralateral breast cancer risk among US women with breast cancer in a general community setting. *JAMA Oncol.*.

[2] Decensi A, Sun Z, Guerrieri-Gonzaga A (2014). Bone mineral density and circulating biomarkers in the BIG 1–98 trial comparing adjuvant letrozole, tamoxifen and their sequences. *Breast Cancer Res. Treat.*.

[3] Freedman AN, Yu B, Gail MH (2011). Benefit/risk assessment for breast cancer chemoprevention with raloxifene or tamoxifen for women age 50 years or older. *J. Clin. Oncol.*.

[4] Chlebowski RT, Schottinger JE, Shi J, Chung J, Haque R (2015). Aromatase inhibitor, tamoxifen and endometrial cancer in breast cancer survivors. *Cancer*.

[5] Sestak I, Kealy R, Nikoloff M (2012). Relationships between CYP2D6 phenotype, breast cancer and hot flushes in women at high risk of breast cancer receiving prophylactic tamoxifen: results from the IBIS-I trial. *Br. J. Cancer*.

[6] Jordan VC (2007). New insights into the metabolism of tamoxifen and its role in the treatment and prevention of breast cancer. *Steroids*.

[7] Etienne MC, Milano G, Fischel JL (1989). Tamoxifen metabolism: pharmacokinetic and *in vitro* study. *Br. J. Cancer*.

[8] Desta Z, Ward BA, Soukhova NV, Flockhart DA (2004). Comprehensive evaluation of tamoxifen sequential biotransformation by the human cytochrome p450 system *in vitro:* prominent roles for CYP3A and CYP2D6. *J. Pharmacol. Exp. Ther.*.

[9] Johnson MD, Zuo H, Lee K-H (2004). Pharmacological characterization of 4-hydroxy-*N*-desmethyl tamoxifen, a novel active metabolite of tamoxifen. *Breast Cancer Res. Treat.*.

[10] Madlensky L, Natarajan L, Tchu S (2011). Tamoxifen metabolite concentrations, CYP2D6 genotype and breast cancer outcomes. *Clin. Pharmacol. Ther.*.

[11] Stearns V, Johnson MD, Rae JM (2003). Active tamoxifen metabolite plasma concentrations after coadministration of tamoxifen and the selective serotonin reuptake inhibitor paroxetine. *J. Natl Cancer Inst.*.

[12] Hicks JK, Gaedigk A, Swen JJ (2014). Challenges in CYP2D6 phenotype assignment from genotype data: a critical assessment and call for standardization [Internet]. *Curr. Drug Metab.*.

[13] Kiyotani K, Mushiroda T, Zembutsu H, Nakamura Y (2013). Important and critical scientific aspects in pharmacogenomics analysis: lessons from controversial results of tamoxifen and CYP2D6 studies. *J. Hum. Genet.*.

[14] Harris LN, Ismaila N, McShane LM (2016). Use of biomarkers to guide decisions on adjuvant systemic therapy for women with early-stage invasive breast cancer: american society of clinical oncology clinical practice guideline. *J. Clin. Oncol.*.

[15] Cronin-Fenton DP, Damkier P, Lash TL (2014). Metabolism and transport of tamoxifen in relation to its effectiveness: new perspectives on an ongoing controversy. *Future Oncol.*.

[16] Lien EA, Ueland PM, Solheim E, Kvinnsland S (1987). Determination of tamoxifen and four metabolites in serum by low-dispersion liquid chromatography. *Clin. Chem.*.

[17] Lien EA, Solheim E, Kvinnsland S, Ueland PM (1988). Identification of 4-hydroxy-*N*-desmethyltamoxifen as a metabolite of tamoxifen in human bile. *Cancer Res.*.

[18] MacCallum J, Cummings J, Dixon JM, Miller WR (1997). Solid-phase extraction and high-performance liquid chromatographic determination of tamoxifen and its major metabolites in breast tumour tissues. *J. Chromatogr. B. Biomed. Sci. App.*.

[19] Lee K-H, Ward BA, Desta Z, Flockhart DA, Jones DR (2003). Quantification of tamoxifen and three metabolites in plasma by high-performance liquid chromatography with fluorescence detection: application to a clinical trial. *J. Chromatogr. B*.

[20] Rama Raju KS, Taneja I, Singh SP (2015). Simultaneous determination of centchroman and tamoxifen along with their metabolites in rat plasma using LC-MS/MS. *Bioanalysis*.

[21] Drooger JC, Jager A, Lam M-H (2015). Development and validation of an UPLC–MS/MS method for the quantification of tamoxifen and its main metabolites in human scalp hair. *J. Pharm. Biomed. Anal.*.

[22] Tré-Hardy M, Capron A, Antunes MV, Linden R, Wallemacq P (2016). Fast method for simultaneous quantification of TAM and metabolites in dried blood spots using an entry level LC–MS/MS system. *Clin. Biochem.*.

[23] Gjerde J, Kisanga ER, Hauglid M, Holm PI, Mellgren G, Lien EA (2005). Identification and quantification of tamoxifen and four metabolites in serum by liquid chromatography–tandem mass spectrometry. *J. Chromatogr. A*.

[24] Teunissen SF, Jager NGL, Rosing H, Schinkel AH, Schellens JHM, Beijnen JH (2011). Development and validation of a quantitative assay for the determination of tamoxifen and its five main Phase I metabolites in human serum using liquid chromatography coupled with tandem mass spectrometry. *J. Chromatogr. B*.

[25] Aranda EO, Esteve-Romero J, Rambla-Alegre M, Peris-Vicente J, Bose D (2011). Development of a methodology to quantify tamoxifen and endoxifen in breast cancer patients by micellar liquid chromatography and validation according to the ICH guidelines. *Talanta*.

[26] Binkhorst L, Mathijssen RHJ, Ghobadi Moghaddam-Helmantel IM (2011). Quantification of tamoxifen and three of its Phase I metabolites in human plasma by liquid chromatography/triple-quadrupole mass spectrometry. *J. Pharm. Biomed. Anal.*.

[27] Antunes MV, Rosa DD, Viana T dos S, Andreolla H, Fontanive TO, Linden R (2013). Sensitive HPLC–PDA determination of tamoxifen and its metabolites *N*-desmethyltamoxifen, 4-hydroxytamoxifen and endoxifen in human plasma. *J. Pharm. Biomed. Anal.*.

[28] Arellano C, Allal B, Goubaa A, Roché H, Chatelut E (2014). An UPLC–MS/MS method for separation and accurate quantification of tamoxifen and its metabolites isomers. *J. Pharm. Biomed. Anal.*.

[29] Dahmane E, Boccard J, Csajka C (2014). Quantitative monitoring of tamoxifen in human plasma extended to 40 metabolites using liquid-chromatography high-resolution mass spectrometry: new investigation capabilities for clinical pharmacology. *Anal. Bioanal. Chem.*.

[30] Fotoohi AK, Karim H, Lafolie P (2016). Pronounced interindividual but not intraindividual variation in tamoxifen and metabolite levels in plasma during adjuvant treatment of women with early breast cancer. *Ther. Drug Monit.*.

[31] Murphy C, Fotsis T, Pantzar P, Adlercreutz H, Martin F (1987). Analysis of tamoxifen, *N*-desmethyltamoxifen and 4-hydroxytamoxifen levels in cytosol and KCl-nuclear extracts of breast tumours from tamoxifen treated patients by gas chromatography-mass spectrometry (GC-MS) using selected ion monitoring (SIM). *J. Steroid Biochem.*.

[32] Dahmane E, Mercier T, Zanolari B (2010). An ultra performance liquid chromatography–tandem MS assay for tamoxifen metabolites profiling in plasma: first evidence of 4′-hydroxylated metabolites in breast cancer patients. *J. Chromatogr. B*.

[33] Antunes MV, Rosa DD, Viana T dos S, Andreolla H, Fontanive TO, Linden R (2013). Sensitive HPLC–PDA determination of tamoxifen and its metabolites *N*-desmethyltamoxifen, 4-hydroxytamoxifen and endoxifen in human plasma. *J. Pharm. Biomed. Anal.*.

[34] Johänning J, Heinkele G, Precht JC (2015). Highly sensitive simultaneous quantification of estrogenic tamoxifen metabolites and steroid hormones by LC-MS/MS. *Anal. Bioanal. Chem.*.

[35] FDA guidance for industry: analytical procedures and methods validation for drugs and biologics - ECA academy [Internet]. https://www.gmp-compliance.org/guidelines/gmp-guideline/fda-guidance-for-industry-analytical-procedures-and-methods-validation-for-drugs-and-biologics.

[36] Viswanathan CT, Bansal S, Booth B (2007). Quantitative bioanalytical methods validation and implementation: best practices for chromatographic and ligand binding assays. *Pharm. Res.*.

[37] Validation of analytical procedures: text and methodology: ICH [Internet]. http://www.ich.org/products/guidelines/quality/quality-single/article/validation-of-analytical-procedures-text-and-methodology.html.

[38] Nowatzke W, Woolf E (2007). Best practices during bioanalytical method validation for the characterization of assay reagents and the evaluation of analyte stability in assay standards, quality controls, and study samples. *AAPS J.*.

[39] Wang Z, Jiang J, Hu P, Zhao Q (2017). Development and validation of a UPLC–MS/MS method for simultaneous determination of fotagliptin and its two major metabolites in human plasma and urine. *Bioanalysis*.

[40] Jager NG, Rosing H, Schellens JH, Beijnen JH (2014). Determination of tamoxifen and endoxifen in dried blood spots using LC-MS/MS and the effect of coated DBS cards on recovery and matrix effects. *Bioanalysis*.

[41] Teunissen SF, Rosing H, Koornstra RHT (2009). Development and validation of a quantitative assay for the analysis of tamoxifen with its four main metabolites and the flavonoids daidzein, genistein and glycitein in human serum using liquid chromatography coupled with tandem mass spectrometry. *J. Chromatogr. B*.

[42] Hughes NC, Bajaj N, Fan J, Wong EY (2009). Assessing the matrix effects of hemolyzed samples in bioanalysis. *Bioanalysis*.

